# Preparing Poly (Lactic-co-Glycolic Acid) (PLGA) Microspheres Containing Lysozyme-Zinc Precipitate Using a Modified Double Emulsion Method

**Published:** 2011

**Authors:** Nastaran Nafissi Varcheh, Vera Luginbuehl, Reza Aboofazeli, Hans Peter Merkle

**Affiliations:** a *Department of Pharmaceutical Biotechnology, School of Pharmacy, Shaheed Beheshti University of Medical Sciences, Tehran, Iran.*; b *Institute of Pharmaceutical Sciences, ETH Zurich, Zurich, Switzerland.*; c *Department of Pharmaceutics, School of Pharmacy, Shaheed Beheshti University of Medical Sciences, Tehran, Iran.*

**Keywords:** Lysozyme, Microencapsulation, Poly (lactic-co-glycolic acid), Protein micronization, Zinc complexation

## Abstract

Lysozyme, as a model protein, was precipitated through the formation of protein-Zn complex to micronize for subsequent encapsulation within poly (lactic-co-glycolic acid) (PLGA) microspheres. Various parameters, including pH, type and concentration of added salts and protein concentration, were modified to optimize the yield of protein complexation and precipitation. The resulting protein particles (lysozyme-Zn complex as a freshly prepared suspension or a freeze-dried solid) were then loaded into PLGA (Resomer^®^ 503H) microspheres, using a double emulsion technique and microspheres encapsulation efficiency and their sizes were determined. It was observed that salt type could significantly influence the magnitude of protein complexation. At the same conditions, zinc chloride was found to be more successful in producing pelletizable lysozyme. Generally, higher concentrations of protein solution led also to the higher yields of complexation and at the optimum conditions, the percentage of pelletizable lysozyme reached to 80%. Taking advantage of this procedure, a modified technique for preparation of protein-loaded PLGA microspheres was established, although it is also expected that this technique increases the protein drugs stabilization during the encapsulation process.

## Introduction

Although the production of various proteins has been possible due to the recent advances in biotechnology and bioengineering, their stability inside the pharmaceuticals and during delivery is one of the major challenges for successful administration. Many available peptide and protein drugs are characterized by short biological half-lives. They are easily degraded by enzymes and poorly pass through biological barriers due to poor diffusivity and low partition coefficient (1). Nowadays, the assessment of protein stability in delivery systems is increasingly being integrated into research programs. Among the various means usually employed, nano- or microspheres made of poly (lactic-co-glycolic acid) (PLGA) has gained some popularity, mainly due to their tissue compatibility and biodegradability (2-6). 

Protein encapsulation and preparation of PLGA microspheres are generally performed using water-in-oil-in-water (w_1_/o/w_2_) double emulsion technique. In this process, an active ingredient is first dissolved in an aqueous phase (w_1_) which is then emulsified in an organic solvent of a polymer to make a primary w_1_/o emulsion. This primary emulsion is further mixed in an emulsifier-containing aqueous solution (w_2_) to make a w_1_/o/w_2_ double emulsion. The removal of the solvent leaves microspheres in the aqueous continuous phase, making it possible to collect them by filtering or centrifuging (7-9).

During microencapsulation, proteins are extremely subjected to several stress factors. Emulsifying a protein solution in an organic phase containing polymer could result in protein denaturation, probably due to the aggregation at the water/organic solvent interface, adsorption to the polymer and unfolding. Therefore, protein stability still remains one of the most important obstacles during their successful incorporation in particulate systems (3, 8, 10, 11). An alternative encapsulation procedure is the solid-in-oil-in-water (s/o/w) technique in which dehydrated protein powders are being used to create a suspension in an organic solvent, followed by emulsification in an aqueous solution to allow microsphere formation and hardening. This procedure eliminates the w_1_/o emulsion step and because of the absence of water/organic solvent interface, it might in turn increase protein stability within encapsulation procedure (3, 12-14). Nevertheless, one of the problems hampering the use of s/o/w technique is the low encapsulation efficiency (15). It is notable that encapsulation yield is an important parameter for cost-efficient production of microspheres containing expensive pharmaceutical proteins. Particle characteristics of the solid protein powder have been introduced as determinant factors in this regard. The smaller the particles sizes, the higher the encapsulation efficiency. Therefore, particle design is a key to the development of protein delivery systems (16). 

The increasing demand for protein particles suitable for drug delivery is currently met using a number of techniques, such as lyophilization (17), milling process and spray drying (18), supercritical fluid technology (19), and more recently, spray-freeze drying (SFD) (20, 21). 

Previous research revealed the possibility of protein micronization in the nm range through a simple and fast precipitation procedure using some metal salts. In addition, it is evidenced that the presence of divalent metal ions (such as Zn^2+^) can enhance the stability of the protein in the required conformation for biological activity (22, 23). Metal affinity protein precipitation has been described as a polymerization process in which multivalent metals serve as cross-linking agents between the protein molecules. The important parameters involved in complexation are the affinity of protein for the metal, the available metal coordination sites, protein and metal concentrations, and pH of the solution (23). Zinc-induced precipitation is commonly used in biochemistry to fractionate proteins from complex mixtures (24). In the drug delivery field, zinc precipitates have been used, for example, to enhance the stability and the duration of recombinant insulin action (22) to prepare sustained release formulation of therapeutic proteins (23) and to precipitate purified human growth hormone as an initial step in preparing controlled release formulations of protein in biodegradable microspheres (25).

The aim of the present study was to apply protein-Zn particles for microsphere preparation. Lysozyme was employed as a model protein. Primarily, the main focus was to obtain an optimum lysozyme-Zn precipitation yield as much as possible by changing various process parameters. Based on an optimum protocol for protein complexation, the resulting protein particles were then loaded into PLGA microspheres, using a double emulsion technique to ensure the protein stability upon encapsulation. The size of the microspheres and the encapsulation efficiency were also determined in this study.

## Experimental

*Materials*

Resomer^®^ 503H, a grade of PLGA with a lactide to glycolide ratio of 50:50, was purchased from Boehringer Ingelheim) Ingelheim, Germany). Lysozyme of hen egg-white was obtained from Fluka (Buchs, Switzerland). The chemicals used were Physiogel^®^ (succinylated gelatin) 4% from B. Braun Medical AG (Emmenbrücke, Switzerland); dichloromethane (DCM) from Scharlau (Tagertwil, Switzerland) and polyvinyl alcohol (PVA) 88% hydrolyzed (Mowiol ^®^ 8-88) from Kuray Co. (Germany). All other substance were of analytical grade and obtained from Fluka (Buchs, Switzerland). Ultrapure water obtained from a Milli-Q purification system (Millipore Corp., Bedford, MA, USA) was used to prepare all solutions.

*Methods*

*Preparation of Zn-Lysozyme complex*

Metal salt-induced precipitation of lysozyme was performed similar to a previously published method (23). Briefly, aqueous lysozyme solution (10 to 75 mg/mL) containing 1% w/v PVA was mixed with zinc salt solution (chloride or acetate) to reach a protein : Zn (Pr : Zn) molar ratio of 1 : 10 to 1 : 100. pH was subsequently adjusted by adding 1 N sodium hydroxide solution. The samples were incubated for 1 h while stirred magnetically at room temperature. When necessary, the prepared complex was freeze-dried following the immersion in liquid nitrogen.

*Determination of lysozyme content in pellets*

The resulting lysozyme-Zn suspensions were centrifuged at 15800 g for 10 min (Eppendorf centrifuge 5415C, Hamburg, Germany). The supernatant of each sample was properly diluted with 0.1 N HCl or acetic acid (depending on the type of zinc salt used for complexation) and assayed spectrofluorimetrically. Meanwhile, the content of lysozyme in the pellets was also determined following the cleavage of pr-Zn complex through dissolving in 90% v/v acetic acid. 

All fluorescence monitoring were done at 280 nm excitation and 335 to 345 nm emission wavelengths (Cary Eclipse Fluorimeter, Varian, Switzerland). The emission wavelength was selected by the initial scanning of lysozyme in the analysis media (water, HCL solution or acetic acid solution) and the individual calibration curves for the concentrations of 5 to 50 μg/mL were constructed for the protein determination in each medium. 

The percentage of pelletable lysozyme, lysozyme _pellet_, was calculated as follows:

${\%\mathrm{Lysozyme}}_{\mathrm{pellet}}=\frac{{\mathrm{Lysozyme}}_{\mathrm{total}}-{\mathrm{Lysoztyme}}_{\mathrm{supernatant}}}{{\mathrm{Lysozyme}}_{\mathrm{total}}}\times 100\%$

In this formula, lysozyme_total _and lysozyme_ernatant_ are the lysozyme content in the preparation based on the amount of lysozyme stock solution used and the lysozyme content in the supernatant, respectively. 

*Preparation of microspheres*

Protein-loaded microspheres were prepared by a solvent extraction technique. Briefly, a sample of freeze-dried protein-Zn complex (for L_1_ microspheres) or freshly prepared suspension (for L_2 _and L_3_ microspheres) containing 2 mg of lysozyme was added to 100 mg of PLGA dissolved in 2 mL anhydrous dichloromethane. The mixture was then sonicated with a CV18 3248 probe and Vibracell pump (Sonics Materials, Danbury, USA) at 50 W for 30 sec. The resultant dispersion was introduced into 50 mL of 5% w/v pre-cooled (10°C) aqueous poly vinyl alcohol solution (PVA-solution) under mechanical mixing (IKA, Germany) at 500 rpm for 2 min. For solvent extraction, the mixture was subsequently diluted with 500 mL of 1% w/v pre-cooled (10°C) aqueous PVA solution and stirred magnetically for 6 h. The obtained microspheres were collected on a 0.45 μm cellulose acetate membrane filter and washed with 500 mL deionized water at room temperature. Finally, the microspheres were dried under reduced pressured (20 mbar) at room temperature overnight. Blank microspheres (L_B_) were prepared with the same method in the absence of protein. For the preparation of L_3_ microspheres, Physiogel^® ^was also added to the protein suspension in the volume ratio of 3 to 1. 

*Particle size analysis*

Size distribution of microspheres was analyzed by dispersing the particles in an aqueous solution of Tween-20^®^ (0.1% v/v). The measurements were carried out by laser light scattering (Malvern Mastersizer X Ver.2.19, Malvern Instruments, UK).

*Determination of lysozyme content*

To determine the lysozyme content in the microspheres, a known weight of vacuum-dried microspheres was taken and dissolved in a certain volume of 90% v/v acetic acid solution. The protein content was then quantified by fluorescence spectroscopy as mentioned earlier.

*Electrophoresis*

Sodium dodecyl sulfate polyacrylamide gel electrophoresis analysis (SDS-PAGE analysis) was performed on microspheres by dispersing 5 mg of them in the sample buffer followed by incubation for 1 h at room temperature.

## Results and Discussion

Lysozyme, a 14 KDa monomeric globular protein with a size similar to many therapeutically effective proteins like interferons and interleukins, was used as a model protein in this study (26, 27). In addition, lysozyme is easily available and well-characterized. Since the stability of the encapsulated protein was not included in the current research, the inhibitory effect of zinc ion on lysozyme activity was not considered to be evaluated.

**Figure 1:**
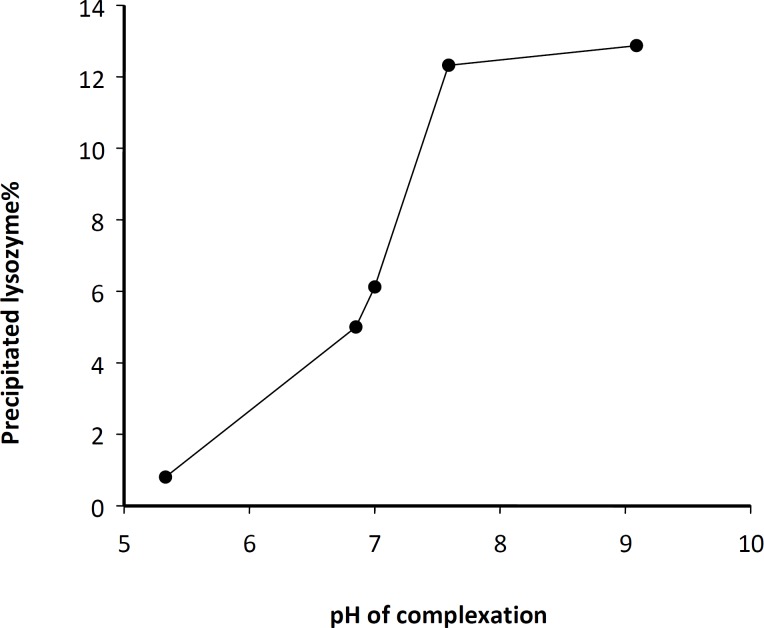
pH dependency of lysozyme-Zn complex formation for a typical experiment condition (initial protein stock solution: 10 mg/mL; Pr : Zn acetate molar ratio, 1: 20).

In order to obtain a high yield of complexation and precipitation, various process parameters were evaluated through classified experiments. It has been shown that the precipitation of a hirudin protein in the pellet is a function of the added amount of NaOH (23). Therefore, the influence of pH on lysozyme-Zn precipitation was investigated. Figure 1 shows the percentage of pelletable lysozyme following the complexation as a function of pH. As depicted, the amount of pelletable lysozyme was highly dependent on the pH of medium during the complex formation. However, it was also observed that similar to hirudin (23), the pH dependency of lysozyme complexation decreases at higher zinc concentrations. According to some previous reports, the adsorption rate at the water/organic solvent interface may increase and the enzyme activity may decrease as the pH approaches the isoelectric (IEP) point of lysozyme (IEP = 11.2) (28). Thus, pH of 7.4 was selected for complexation and further experiments.

**Figure 2:**
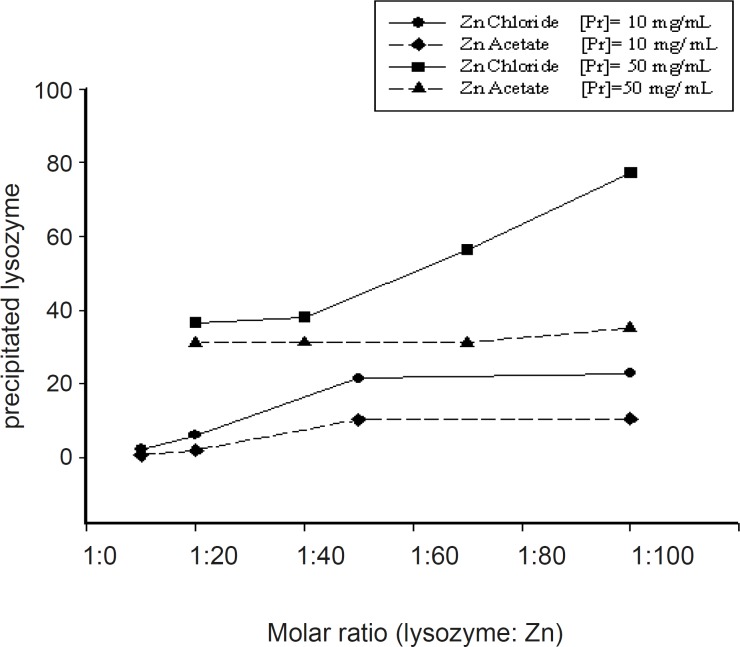
Influence of the zinc salt type on the percentage of lysozyme in the pellet (initial protein stock solution: 10 mg/mL; pH of complexation medium: 7.4).

Salt type was the other studied parameter which significantly influenced the magnitude of protein complexation. The precipitation of lysozyme with chloride and acetate salts of zinc resulted in different outcomes. The data obtained by two various concentrations of protein (10 and 50 mg/mL) using acetate or chloride salts of zinc at various Pr : Zn molar ratios were presented in Figure 2.

**Figure 3:**
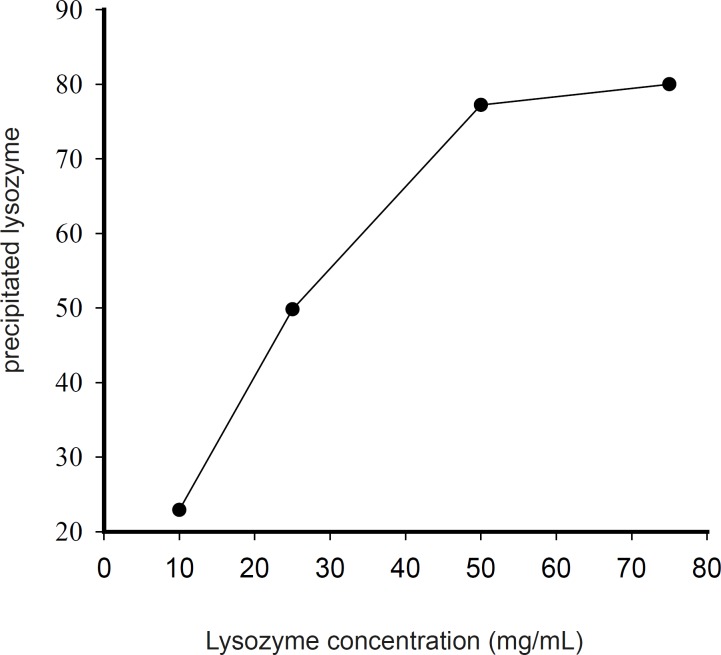
Influence of lysozyme concentration on the percentage of precipitated protein at pH 7.4 and the molar ratio of 1 : 100 lysozyme to zinc chloride).

 Generally, it seems that at the same conditions, zinc chloride was more successful in producing pelletable lysozyme. Higher concentrations of protein solution led to higher yields of complexation (Figure 3). 

By applying a 75 mg/mL lysozyme solution and chloride salt of zinc at Pr : Zn molar ratio of 1 : 100 at pH of 7.4, the percentage of precipitated lysozyme reached nearly 80%. An increase of lysozyme : Zn molar ratio from 1 : 100 to 1 : 250 did not have any significant effect on the percentage of the precipitated protein. Preparation of higher concentration of protein solution was prohibited because of its solubility limit. Thus, 75 mg/mL lysozyme solution and Pr : Zn molar ratio of 1 : 100 were selected for further studies.

In the next phase, lysozyme-Zn complex as a freshly prepared suspension or a freeze-dried solid was encapsulated within the PLGA polymer by a double emulsion method. The characteristics of all prepared microspheres were shown as Table 1. 

**Table 1:** Characteristics of the prepared microspheres (n = 3).

	**Microspheres formulation**	**Properties of microspheres**	
**Code**	**Protein phase**	**Particle size (μm)** Mean ± SD (RSD%)*	**protein% loading Mean ± SD (RSD%)**
**L**_1_	Lyophilized lysozyme-Zn complex	45.74 ± 3.19 (6.97)	3.53 ± 0.8 (23.05)
**L**_2_	Freshly-prepared suspension of lysozyme-Zn complex	36.39 ± 0.79 (2.18)	16.56 ± 1.0 (6.05)
**L**_3_	Freshly-prepared suspension of lysozyme-Zn complex + Physiogel^®^	36.45 ± 0.76 (2.10)	61.74 ± 1.45 (2.35)
**L**_B_	Without protein	33.90 ± 0.36 (1.06)	-

* RSD%: Relative standard deviation

The particle size, was expressed as volume mean diameter ± SD (in μm) of values collected from three different batches. The mean particle size of L_1_-L_3_ microspheres with blank microspheres were significantly different (p < 0.05). Post-hoc tests demonstrated non-significant differences between mean particle sizes of L_2 _and L_3_ (p > 0.05). The differences in mean particle sizes and also the loading percentages between L_1 _microspheres and other formulations were all significant. Although it has been reported that a solid-state protein retains its activity in organic conditions (10), larger particle size and lower protein loading of L_1_ microspheres can be attributed to the observed agglomeration of protein-Zn particles upon lyophilization. Therefore, it was concluded that drying the micronized particles of lysozyme-Zn, at least in the applied conditions of this study, could be harmful for micronization and encapsulation procedures. Since lyophilization itself generates a variety of stresses to denaturate proteins, in order to protect a protein from denaturation (cryoprotection) and/or dehydration (lyoprotection), some stabilizers may be necessary. On the other hand, the design of a lyophilization cycle is a critical parameter and the development of a lyophilized protein product usually takes an enormous amount of time and effort (17). Therefore, if direct encapsulation of a protein-Zn suspension into PLGA polymers results in the production of desirable microspheres, this technique could be considered as an alternative method to the previously reported protocols.

The real loading percentage (w/w) which corresponds to the amount of lysozyme effectively entrapped in a given amount of microspheres, was determined by degrading the particles using a 90% v/v acetic acid solution and assaying the lysozyme content. At this condition, there was no interference in the absorbance reading due to the presence of polymer or any other substance. All experiments were performed in triplicate and the results were reported as mean ± SD (Table 1). Statistical analysis based on the one-way ANOVA indicated significant differences among the loading percentages of various lysozyme-Zn loaded microspheres (p < 0.05).

Lysozyme microencapsulation efficiency increased greatly when Physiogel was added to the aqueous lysozyme-Zn dispersion prior to microencapsulation (L_3_ microspheres). This observation may be related to an increase in the viscosity of the inner aqueous phase, an alteration in interfacial tension and/or an improvement in the stability of the first emulsion during microencapsulation. Also, there is an evidence that adding the excipients to the inner aqueous phase that compete with the water/organic solvent interface, can prevent emulsification-induced denaturation and aggregation (11). Interestingly, no lysozyme aggregates were detected in protein-Zn-loaded microspheres by SDS-PAGE (data not shown). It is expected that complexation of protein with zinc decreases the adverse effects at the organic solvent/water interface by removing (L_1_ microspheres) or by shielding the protein from it (L_2_ and L_3 _microspheres). 

## Conclusion

A new technique for preparation of protein-loaded PLGA microspheres was established, which involves two independent but consecutive processes, *i.e. *micronization and microencapsulation. For incorporation of therapeutic proteins or peptides into polymers by using this new technique, the optimum conditions of pH, protein concentration, type and the concentration of zinc salt should individually be determined. In the present study, zinc has been essentially applied for protein micronization; however, it seems to play an important role in protein stabilization during the encapsulation process.
